# Therapies for cutaneous squamous cell carcinoma in recessive dystrophic epidermolysis bullosa: a systematic review of 157 cases

**DOI:** 10.1186/s13023-024-03190-1

**Published:** 2024-05-21

**Authors:** Austin Hwang, Andie Kwon, Corinne H. Miller, Antonia Reimer-Taschenbrecker, Amy S. Paller

**Affiliations:** 1 Departments of Dermatology and Pediatrics, Northwestern University Feinberg School of Medicine, 676 North St Clair Street, Suite 1600, Chicago, IL 60611 USA; 2 Department of Dermatology, University of Freiburg Medical Center, Freiburg, Germany; 3 Galter Health Sciences Library & Learning Center, Northwestern University Feinberg School of Medicine, Chicago, IL USA

**Keywords:** Anti-EGFR therapy, Chemotherapy, Epidermolysis bullosa, Immunotherapy, Radiotherapy, Recessive dystrophic epidermolysis bullosa, Skin cancer, Squamous cell carcinoma, Patient survival, Therapy, Treatment

## Abstract

**Background:**

Invasive cutaneous squamous cell carcinomas (cSCC) are a leading cause of death in recessive dystrophic epidermolysis bullosa (RDEB), a rare blistering genodermatosis. Outcomes of RDEB-cSCC therapies have primarily been described in case reports. Systematic studies are scarce. This systematic review aims to assess the pathophysiology, clinical characteristics, and outcomes of RDEB-cSCCs, with a focus on results and mechanisms of recent immunotherapies and anti-EGFR treatments.

**Results:**

A systematic literature search of epidermolysis bullosa and cSCC was performed in February 2024, using PubMed, Embase, Cochrane Central Register of Controlled Trials, ClinicalTrials.gov, and EudraCT databases. Cases with administration of systematic therapies and unpublished outcomes regarding death were tracked with corresponding authors. Data extraction and risk of bias assessment was performed by two independent reviewers. Of 1132 references in the original search, 163 relevant articles were identified, representing 59 case reports, 7 cohort studies, 49 abstracts, 47 in-vitro/in-vivo experiments, and 1 bioinformatic study. From these, 157 cases of RDEB-cSCCs were included. The majority of RDEB-cSCCs were well-differentiated (64.1%), ulcerated (59.6%), and at least 2 cm in size (77.6%), with a median age at diagnosis of 30 years old (range 6–68.4). Surgery was the primary form of treatment (*n* = 128), followed by chemotherapy and radiotherapy. Anti-EGFR therapy and immunotherapy was also reported beginning in 2009 and 2019, respectively. Survival time from first cSCC diagnosis to death was available in 50 cases. When stratified by their treatment regimen, median survival time was 1.85 years (surgery + chemotherapy, *n* = 6), 2 years (surgery only, *n* = 19), 4.0 years (+ anti-EFGR therapy, *n* = 10), 4 years (surgery + radiotherapy, n = 9), 4.6 years (+ immunotherapy, *n = *4), and 9.5 years (surgery + chemotherapy + radiotherapy; *n = *2). Treatment-related adverse events were primarily limited to impaired wound healing for immunotherapies and nausea and fatigue for anti-EGFR therapies.

**Conclusions:**

Despite the challenges of a limited sample size in a rare disease, this systematic review provides an overview of treatment options for cSCCs in RDEB. When surgical treatment options have been exhausted, the addition of immunotherapy and/or anti-EGFR therapies may extend patient survival. However, it is difficult to attribute extended survival to any single treatment, as multiple therapeutic modalities are often used to treat RDEB-cSCCs.

**Supplementary Information:**

The online version contains supplementary material available at 10.1186/s13023-024-03190-1.

## Background

Recessive dystrophic epidermolysis bullosa (RDEB) is a rare inherited skin blistering disorder, characterized by a marked deficiency of functional collagen VII [1]. The leading cause of death in adults with RDEB is cutaneous squamous cell carcinoma (cSCC), with an age-related increasing cumulative risk of developing at least one cSCC (7.5% at age 20 years, 52% at 30 years, and 80% at 45 years) and subsequent mortality (38.7% by age 35 years, 70% by 40 years, and 78.7% by 55 years) [2].

The pathogenesis of RDEB-cSCCs has not been fully elucidated. However, existing literature suggests that pro-inflammatory cytokines, APOBEC family proteins, and proteins involved in fibrosis play a role in its aggressive nature relative to conventional UV-induced cSCC [3–5]. Furthermore, RDEB-cSCCs often occur on the extremities in photo-protected areas of chronic ulceration and fibrosis [6].

Surgical management by wide, local excision or amputation is considered first-line therapy for RDEB-cSCCs. However, determining tumor margins is challenging due to the background of inflammation and scarring in RDEB [7]. Other therapies such as chemotherapy, radiotherapy, immunotherapy, and anti-epidermal growth factor receptor (EGFR) may also be recommended for palliative care or for advanced and/or metastatic SCCs [7]. However, evidence is limited to a handful of case reports and series.

This systematic review aims to summarize clinical advancements in the treatment of RDEB-cSCCs [8, 9] with a focus on results and mechanisms related to EGFR and PD-1 inhibitor therapies. Our review additionally presents data on patient survival after various therapeutic modalities.

## Results

**Fig. 1:**
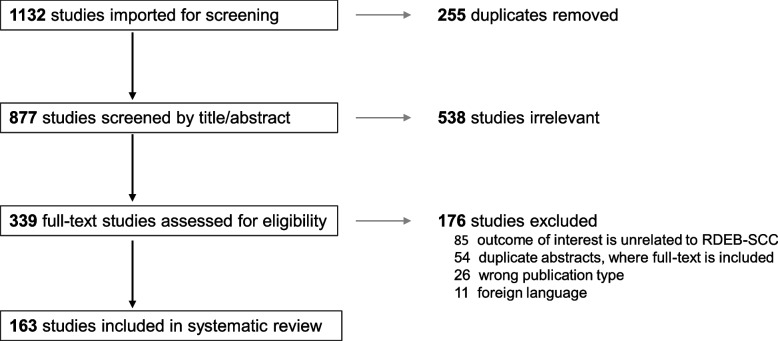
Systematic review PRISMA diagram

Of 1132 references in the original search, 163 relevant articles were identified, representing 59 case reports, 7 cohort studies, 49 abstracts, 47 in-vitro/in-vivo experiments, and one bioinformatic study (Fig. 1). From this, 157 cases of RDEB with at least one cSCC were included. Among them, 76 were classified as RDEB-severe (RDEB-S), 13 RDEB-intermediate (RDEB-I), 1 RDEB-inversa, and 1 RDEB-pruriginosa. The remaining 66 cases were of undefined RDEB clinical subtype. The diagnosis of RDEB was made using multiple methods, including clinical diagnosis only (*n = *23), genetic analysis (*n = *39), immunohistochemistry (*n = *18), and electron microscopy (*n = *16); 70 cases did not report diagnostic techniques.

### Pathophysiology

While RDEB-cSCC pathogenesis is not well understood, many theories attribute the aggressive nature of RDEB-cSCCs to factors involving altered wound healing processes, genetic differences, and impaired immune responses. The absence of type VII collagen, inherent to RDEB, directly interferes with wound healing by enhancing keratinocyte migration and upregulating tumorigenesis and angiogenesis through high levels of TGF-β signaling [4, 5, 10]. Increased TGF-β expression is hypothesized to cause a stroma altered in its organization, driving tumor progression through mechanosensing signaling by β1 integrin, activated focal adhesion kinase (FAK), and phosphoinositide 3-kinase (PI3K) [4].

Specific mutations in *NOTCH1/2/4*, *TP53*, and *MYC*, along with the activation of TGF-β receptor signaling pathways, may distinguish the clinical behavior of RDEB-cSCCs [11–13]. In contrast to conventional cSCCs, UV damage does not play a significant role in RDEB-cSCC pathogenesis. Instead, driver mutations in RDEB-cSCCs are generated endogenously by high activity rates of APOBEC (apolipoprotein-B mRNA editing enzyme, catalyic polypeptide-like) enzymes that are often observed in chronic wounds [3], accounting for the typical localization of RDEB-cSCCs to chronic wound sites. Peritumoral infiltration by immune cells is also reduced in RDEB skin. In particular, RDEB-cSCCs demonstrated significant reductions in CD3^+^, CD4^+^, and CD68^+^ expression (cells/mm^2^) compared to conventional cSCCs, and CD3^+^, CD4^+^, CD8^+^, and CD20^+^ compared to secondary cSCC (post-burns and post-radiotherapy) [14].

### Clinical presentation

The median age at diagnosis specified in 144 individuals with RDEB and cSCCs was 30 years (interquartile range (IQR) 24–36 years and range, 6–68 years); only 7 were patients younger than 18 years. The majority of RDEB-cSCCs were well-differentiated (59 of 92; 64.1%), ulcerated (31 of 51; 59.6%), and at least 2 cm in size (52 of 67; 77.6%). No sex differences were observed in RDEB as a whole or its severe vs. intermediate subtypes. RDEB-cSCCs often presented in the distal upper and lower extremities with the greatest predilection for the feet (28.3%), shins including knee (21.5%), hands (19.3%), and forearm including elbow (13.0%). The presence of metastases was evaluated in 73 cases (46.5%); of these, 41 cases (56.2%) demonstrated loco-regional and 8 cases (11.0%) visceral metastases. The remaining 24 cases (32.9%) had not developed metastases at the time of reporting. Table 1 presents additional information on the demographic, clinical, and histopathological features of the included cSCCs.

**Table 1:** Patient demographics and clinical features of cSCC in RDEB

		RDEB Total^a^ (%)	RDEB-Sev	RDEB-Int
Sex	Male	72 (45.9)	34	7
	Female	85 (54.1)	42	6
	Ratio (M/F)	0.85	0.81	1.17
Median age at diagnosis years (range), specified in 144 cases (91.7%)		30 (6–68.4)	28.8 (13.4–56)	39 (12–68.4)
Median number of SCCs (range), specified in 142 cases (90.4%)		2 (1–80)	2 (1–44)	2 (1–56)
Location, specified in 99 cases (63.1%)				
	Head/Neck	8 (3.6)	3	3
	Chest	4 (1.8)	1	0
	Abdomen	1 (0.4)	0	0
	Back	5 (2.2)	3	0
	Upper Arm	9 (4.0)	2	0
	Forearm (including elbow)	28 (13.0)	6	0
	Hand	43 (19.3)	10	1
	Hip/Buttocks	4 (1.8)	3	0
	Upper Leg	9 (4.0)	5	0
	Lower Leg (including knee)	48 (21.5)	7	0
	Foot	63 (28.3)	18	2
Clinical Features, specified in 52 cases (33.1%); EB subtype clear in 17 of them	Ulcerated	31 (59.6)	8	1
	Exophytic/Hyperkeratotic	11 (21.2)	5	0
	Verrucous, crusted, or erosive	10 (19.2)	2	1
Size, specified in 45 cases (28.6%)	≤ 2 cm	15 (22.4)	2	1
	2 – 5 cm	21 (31.3)	5	1
	≥ 5 cm	31 (46.3)	7	0
Histopathologic Characteristics, specified in 92 cases (58.6%)	Well-differentiated	59 (64.1)	27	3
	Moderately differentiated	25 (27.2)	19	0
	Poorly differentiated	8 (8.7)	6	0
Metastases, specified in 73 cases (46.5%)	None	24 (32.9)	16	2
	Loco-regional	41 (56.2)	22	0
	Visceral	8 (11.0)	4	0

^a^RDEB subtype was not specified in all sources. In such cases, the individual data was only incorporated into RDEB total. *Sev* Severe, *Int* Intermediate

### Assessment and diagnosis

The diagnosis of RDEB-cSSC is made clinically and histologically. Although not performed routinely, immunohistochemistry for tumor PD-L1 and EGFR expression may predict whether a patient benefits from targeted systemic therapies, such as immunotherapies and anti-EGFR (primarily cetuximuab) treatments. Among patients treated with immunotherapies in our systemic review, immunohistochemistry for PD-L1 or EGFR expression was performed in 11 (52.4%) prior to the initiation of immunotherapy; 10 had no testing performed or did not specify. Predictors of positive response to cetuximab therapy include tumor overexpression of EGFR and absence of mutations in the *KRAS gene* [15, 16]. However, the molecular profile of cSCCs in EB was only been reported in 9 of 21 cases treated with immunotherapy or anti-EGFR therapy [15, 17–22]. In these, EGFR (*n = *7), PD-L1 (*n = *3), and COX-2 (*n = *1) were overexpressed based on immunohistochemistry. *TERT* (*n = *1) and *CTCF* mutations (*n = *1) have been identified by RNA sequencing.

Other unique biomarkers are also overexpressed in RDEB-cSCCs. Serine proteases, C1r, and C1s, were found to be significantly overexpressed in RDEB-associated and invasive sporadic cSCCs, relative to cSCC in situ, actinic keratosis, and normal skin [23]. Cancer-type *SLCO1B3* transcripts were specifically detected in RDEB-cSCC cell lines (*n = *7) and isolated from extracellular vesicles, both in vitro and in the serum of tumor-bearing mice [24]. For assessment of metastases, local and regional assessments were primarily diagnosed using lymph node biopsy (*n = *13) to distinguish inflammation vs. metastasis and magnetic resonance imaging to evaluate extent of tumor invasion (*n = *7). Pathological examination confirmed the presence of axillary, clavicular, or inguinal lymph node metastases in eight cases (53.3%) [25–28], while five cases were attributed to nodal inflammation [25, 29, 30]. Due to limited details in case reports, lymph node biopsies could not be further specified as sentinel nodes or random regional samples; as such, the role of sentinel node biopsies remains unclear in surgical cSCC resections. In five cases, primary cSCCs invaded the muscles (*n = *4) and neurovasculature (*n = *1) [15, 31–34]. In one case, initial imaging raised the suspicion of bone metastases but subsequent biopsy demonstrated osteonecrosis without tumor cells [35].

Distant metastases were evaluated by computed tomography imaging (CT; *n = *16) and positron emission tomography-computed tomography imaging (PET-CT; *n = *26). CT imaging detected metastases in 7 cases (43.8%), in which three showed loco-regional metastases in axillary and/or clavicular lymph nodes [18, 28]. Visceral metastases were detected in the lungs in four cases [17, 32, 36, 37]. In the remaining nine cases, imaging was negative [28, 30, 31, 35, 38–40]. When evaluated by PET-CT imaging, regional metastases were detected in ten cases [15, 18, 28]. In the remaining 16 cases, imaging was negative [20, 22, 28, 41, 42].

## Treatments

### Surgery

Surgical approaches to cSCCs were reported in 131 cases. The most common techniques included excisions (*n = *96; 73.2%), amputations (*n = *29; 22.1%), and Mohs micrographic surgery (*n = *3; 2.3%). Surgical excision was performed in 96 cases and clinical outcomes were described in 31. In nine cases, patients developed loco-regional (*n = *7) or visceral metastasis (*n = *2). In 11 cases, no evidence of recurrence or metastases were observed at follow-up, ranging from 2–36 months (median 6.5 months). In the remaining cases, patients developed more cSCCs and underwent additional therapeutic interventions [43]. Mohs micrographic surgery (MMS) was performed in 3 cases, with only one patient achieving complete response at 16 months of follow-up [44].

Surgical amputations of at least one part of a limb were reported in 29 cases, with surgical sites specified in 23 [45, 46]. Amputations of the lower or upper extremities were performed in 8 and 14 cases, respectively. Both lower and upper distal extremities were amputated in 1 patient. The clinical course of these patients was further described in 14 cases: three developed new cSCCs, three developed loco-regional (*n = *2) or visceral metastases (*n = *1), three achieved complete remission, and five patients were reported to have died, with 3 related to metastasis.

### Chemotherapy

Thirty patients were treated with conventional chemotherapy (*n = *12) or electrochemotherapy (chemotherapy after local electroporation; *n = *18). The clinical outcomes of six patients treated with conventional chemotherapy were further described: one demonstrated disease progression [37], one switched to cetuximab therapy due to poor tolerance of chemotherapy [15], and four died [17, 33, 37, 47]. In comparison, the results of electrochemotherapy are more favorable. By inducing membrane permeability with short, intense electric pulses (electroporation), hydrophilic drugs such as bleomycin can gain direct access to the cytosol and demonstrate higher cytotoxicity by several 100-fold [48]. Among the 18 cases treated with electrochemotherapy, the clinical outcomes of 13 patients were further described: 2 demonstrated disease progression [18, 27], 4 partial response [19, 20, 49, 50], 6 patients complete response [49, 50], and 1 stable disease [27]. Adverse events related to electrochemotherapy were primarily limited to pain, erythema, and ulceration [27, 49, 50].

### Radiotherapy and topical photodynamic therapy

A total of 26 patients were treated with radiotherapy (*n = *24) or topical photodynamic therapy (*n = *2). Of the 24 cases with radiotherapy treatment, the clinical outcomes of 8 patients were reported: one achieved a partial response, one complete response with concurrent chemotherapy of 4 years, two disease progression, and four survived for an additional 3, 6, 7, and 40 months. The total radiation doses ranged from 57 to 70.2 Gy (median 63 Gy) [25, 35, 51, 52]. In one case with topical photodynamic therapy, the patient achieved a complete response with no observable recurrence at a 2-year follow-up [53].

### Immunotherapy

PD-1 inhibitor immunotherapy was used in 11 patients: cemiplimab (*n = *8), nivolumab (*n = *1), and pembrolizumab (*n = *2). One patient concurrently used pembrolizumab and an intralesional oncolytic viral therapy, talimogene laherparepvec (T-VEC). Among those receiving cemiplimab, two achieved complete response and three reported stable disease [22, 27, 34, 54–56], but the clinical outcome of the recent sixth patient remains unknown [28]. Adverse events, particularly mild fatigue and nausea, were commonly reported with cemiplimab therapy. In the single case of nivolumab therapy, treatment was generally well-tolerated apart from fatigue, and the patient has remained in remission off therapy for four months at the time of publication [57]. Of the two patients receiving pembrolizumab, one demonstrated a > 50% size reduction in cSCCs and complete healing of ulcerated areas after 12 months, while the other died from tumor progression [19, 20]. Immune-related thyroiditis was the only adverse event reported with pembrolizumab [20]. In the single case of T-VEC, therapy was administered intralesional to the cervical and axillary lymph node metastases, but tumor progression resulted in patient demise 5 months later [19]. Table 2 summarizes cases of cSCC treated by immunotherapy.

**Table 2:** Immunotherapy treatment of SCC in RDEB patients

Reference	Age (yr)/Sex	RDEB Subtype	Site(s) of SCC under Treatment	Histological Differentiation/tumor size (cm)	Site(s) of Metastases	Treatment	Outcome	Adverse Events
Medek 2019 [19]	33 F	Sev	Forearm	Unknown	Axillary and infraclavicular lymph nodes, in-transit cutaneous and subcutaneous metastases on right upper limb	1. Exc 2. Cet 3. ECT + MTX 4. Pem + T-VEC + Pan	1. Meta 2. RC/RL + Meta 3. PR 4. Death from SCC	(Cet) Impaired wound healing; grade 2 allergic reaction with circulatory collapse, tightness in chest, erythema, fever, and chills
Bruckner 2020 [57]	40 F	N/A	Forearm	Well	Axillary and cervical lymph nodes; chest wall; pathologic fracture of left humerus due to metastatic SCC	1. Cet + RT 2. Amp 3. Niv	1. RC/RL 2. Met 3. SD	(Cet) Impaired wound healing, lymphedema (Niv) Fatigue
Khaddour 2020 [54]	32 M	N/A	Upper arm	Unknown, > 5	Axillary lymph nodes	1. Res 2. 5-FU + MTX + Imi 3. Cem + RT	1. RC/RL 2. Met 3. CR	Fatigue; nausea
O’Sullivan 2020 [34]	28 F	N/A	Chest	Unknown, 2–5	Subcutaneous SCC on right upper chest wall	1. Debulking surgery 2. ECT 3. Cem	1. PD 2. PD 3. SD	None
Piccerillo 2020 [20]	45 F	N/A	Head/neck; Lower leg; Foot	Unknown	N/A	1. Exc 2. ECT 3. Amp 4. Pem	1. RC/RL 2. PR 3. PD 4. PR	(Pem) Immune-related thyroiditis
Reimer 2020 [21]	51 F	Sev	Forearm, Hand, Knee	Unknown	Inguinal lymph nodes	1. Amp 2. Pem 3. Cet	1. Meta 2. PD 3. SD	(Pem), development of new SCCs with reduced PD-1L expression
Robertson 2021 [28]	24^a^ F	Sev	Unknown	Unknown	N/A	1. Exc 2. Imi 3. Systemic retinoid 4. Cem	Unknown	N/A
Duong 2021 [22]	30 F	N/A	Back	Well	N/A	1. Res 2. MTX 3. Cem	1. PD 2. PD 3. SD	Mild fatigue; worsening pruritus
Vasilev 2022 [56]	34 M	N/A	Hand	Moderate, > 5	N/A	1. Exc 2. RT 3. Cem	1. PD 2. PD 3. CR	Pruritus
Trefzer 2023 [55]	16 M	N/A	Forearm, Upper leg, Foot	Well	Inguinal lymph nodes	1. Exc 2. Cem 3. Amp 4. Cet	1. PD 2. PD 3. SD 4. PD	N/A
Trefzer 2023 [55]	36 M	N/A	Head, Upper arm, Forearm, Hand, Foot	Well, < 2	N/A	1. Exc 2. Cem + RT	1. PD 2. SD	N/A

Key: *Amp* Amputation, *Cem* Cemiplimab, *Cet* Cetuximab, *CHT* Unspecified chemotherapy, *CR* Complete response, *ECT* Electrochemotherapy, *Erl* Erlotinib, *Exc* Excision, *5-FU* 5-fluorouracil, *Gem* Gemcitabine, *Imi* Topical imiquimod 5%, *Int* Intermediate RDEB, *Meta* Metastases, *MTX* Methotrexate, *Niv* Nivolumab, *NR* No response, *Pan* Panitumumab, *PD* Progressive disease, *Pem* Pembrolizumab, *PR* Partial response, *Res* Resection, *RC/RL* Local recurrence or relapse, *RT* Radiotherapy, *SD* Stable disease, *Sev* Severe RDEB, *T-VEC* Talimogene laherparepvec

^a^Age at the time of 1st SCC diagnosis, as age at the time of treatment is not reported

### Anti-EGFR therapy

Anti-EGFR therapy was reported in 9 articles, describing 13 patients [6, 15, 17–19, 21, 28, 55–57]. All 13 patients received cetuximab, but one also received panitumumab [19]. Four demonstrated partial response, but later progressed to lung metastases (*n = *2), development of new nodules (*n = *2), or primary tumor progression (*n = *1) [17, 18, 21, 55]. In one patient, marked improvement was initially reported, but the tumor recurred six months after treatment [57]. Stable disease was observed in two patients, noting progression-free survival of at least 3 and 9 months, respectively [15, 19]. Clinical outcomes of the remaining five patients are unknown [6, 28]. Adverse events associated with cetuximab included: impaired wound healing (*n = *2), grade 2 allergic reaction with circulatory collapse, chest tightness, erythema, fever, and chills (*n = *1), vesicular eruption (*n = *1), acneiform folliculitis (*n = *1), and mild dry skin (*n = *1). Table 3 summarizes cases of cSCC treated by anti-EGFR therapy.

**Table 3:** Anti-EGFR treatment of SCC in RDEB patients

Reference	Age (yr)/Sex	RDEB Subtype	Site(s) of SCC under Treatment	Histological Differentiation/ tumor size (cm)	Site(s) of Metastases	Treatment	Outcome	Adverse Events
Arnold 2009 [15]	24 F	Sev	Elbow, Feet	Well/ > 5	Axillary lymph nodes	1. Exc 2. RT 3. CHT 4. Cet	1. RC/RL and Meta 2. PD 3. PR 4. SD	(Cet) acneiform folliculitis
Kim 2013 [17]	26 F	Sev	Hand	Moderately/ > 5	Axillary lymph nodes, Lungs	1. Exc + Amp 2. RT 3. Cet 4. Cet + Gem	1. Meta 2. RC/RL + Meta 3. PD 4. Death from pneumonia	(Cet) Mild skin dryness
Kim 2013 [17]	43 M	Sev	Unknown	Well/ > 5	Axillary lymph nodes, Lungs	1. Exc 2. RT 3. Cet 4. MTX	1. RC/RL 2. PD 3. PD 4. Death from pneumonia	(Cet) Vesicular eruption
Kim 2018 [6]	16^a^	Sev	Unknown	Unknown	Metastasis, unspecified	1. Amp 2. RT 3. Acitretin 4. Cet	Unknown	N/A
Kim 2018 [6]	30^a^	Sev	Unknown	Unknown	Metastasis, unspecified	1. Exc 2. Acetretin 3. CHT 4. Cet	Unknown	N/A
Kim 2018 [6]	39^a^	Int	Unknown	Unknown	Metastasis, unspecified	1. Amp 2. RT 3. Acitretin 4. CHT 5. Cet	Unknown	N/A
Diociaiuti 2019 [18]	15 F	Sev	Upper arm	Well/ > 5 cm	Axillary and clavicular lymph nodes	1. ECT 2. Cet	1. PD 2. Death from SCC	N/A
Diociaiuti 2019 [18]	49	Sev	Lower leg	Poor	Axillary and para-iliac lymph nodes	1. Exc 2. Cet 3. Amp 4. RT	1.RC/RL and Meta 2. PR 3. RX/RL 4. Death from SCC	(Cet) Impaired wound healing
Medek 2019 [19]	33 F	Sev	Forearm	Unknown	Axillary and infraclavicular lymph nodes, in-transit cutaneous and subcutaneous metastases on right upper limb	1. Exc 2. Cet 3. ECT + MTX 4. Pem + T-VEC + Pan	1. Meta 2. RC/RL + Meta 3. PR 4. Death from SCC	(Cet) Impaired wound healing; grade 2 allergic reaction with circulatory collapse, tightness in chest, erythema, fever, and chills
Reimer 2020 [21]	51 F	Sev	Forearm, Hand, Knee	Unknown	Inguinal lymph nodes	1. Amp 2. Pem 3. Cet	1. Meta 2. PD 3. Death	(Pem) development of new SCCs with reduced PD-1L expression
Robertson 2021 [28]	18^a^	Sev	Unknown	Unknown	Loco-regional metastasis, unspecified	1. Exc 2. RT 3. Systemic Retinoid 4. Erl	Unknown	N/A
Robertson 2021 [28]	13^a^	Sev	Unknown	Unknown	Loco-regional metastasis, unspecified	1. Exc 2. Cet	1. RC/RL 2. Death from SCC	N/A
Trefzer 2023 [56]	16 M	N/A	Lower arm, Upper leg, Foot	Well	Inguinal lymph nodes	1. Exc 2. Cem 3. Amp 4. Cet	1. PD 2. PD 3. SD 4. PD	N/A

Key: *Amp* Amputation, *Cet* Cetuximab, *CHT* Unspecified chemotherapy, *ECT* Electrochemotherapy, *Erl* Erlotinib, *Exc* Excision, *5-FU* 5-fluorouracil, *Gem* Gemcitabine, *Int* Intermediate RDEB, *Meta* Metastases, *MTX* Methotrexate, *Niv* Nivolumab, *NR* No response, *Pan* Panitumumab, *PD* Progressive disease, *Pem* Pembrolizumab, *PR* Partial response, *RC/RL* Local recurrence or relapse, *RT* Radiotherapy, *SD* Stable disease, *Sev* Severe RDEB, *T-VEC* Talimogene laherparepvec

^a^Age at the time of 1st SCC diagnosis, as age at the time of treatment is not reported

## Prognosis

**Fig. 2:**
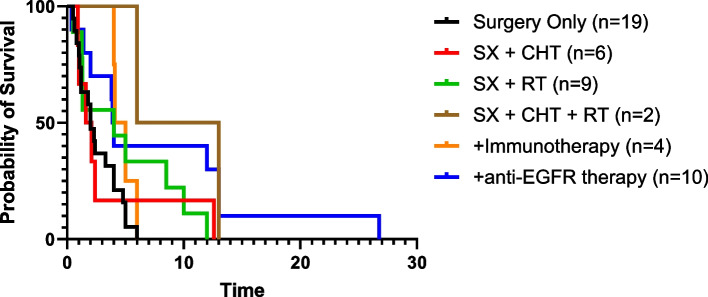
Kaplan–Meier Survival Curve. This curve shows cSCC-specific survival for patients with RDEB based on their therapeutic regimen: Surgery (SX) only (*n = *19); SX + Chemotherapy (CHT) (*n = *6), SX + Radiotherapy (RT) (*n = *9), SX + CHT + RT (*n = *2), + Immunotherapy (*n = *4), and + anti-EGFR therapy (*n = *10)

Given the aggressive nature of RDEB-cSCCs, multiple successive treatment modalities are often used. It is therefore difficult to determine the exact survival benefit of each intervention; however, our systematic review demonstrated that, in most cases, additional interventions after surgical management prolong patient survival. Median survival time was 1.85 years (surgery + chemotherapy, *n = *6), 2 years (surgery only, *n = *19), 4 years (+ anti-EFGR therapy, *n = *10), 4 years (surgery + radiotherapy, *n = *9), 4.6 years (+ immunotherapy, *n = *4), and 9.5 years (surgery + chemotherapy + radiotherapy; *n = *2) (Fig. 2). The median follow-up time from latest treatment could only be determined for systemic treatments: 3.6 years (+ immunotherapy, *n = *1) and 1.7 years (+ anti-EGFR therapy, *n = *3). Furthermore, age at first cSCC diagnosis was not correlated with length of survival (rho = 0.06). Detailed information about patient demographics, histopathological features, and clinical courses can be found in Supplemental Table 1, Additional file 1.

## Discussion

Invasive cSCC is a leading cause of death in patients with RDEB. However, with unique markers such as C1r and C1s, the progression of cSCC can be monitored, and Cancer type-*SLCO1B3* transcripts may be used to detect RDEB-cSCC metastases. Given the aggressive and recurrent presentation of RDEB-cSCCs, often with regional and visceral metastases, C1r, C1s, and Ct-*SLCO1B3* transcripts provide a unique method of monitoring disease progression and determining the appropriate therapeutic intervention.

Wide, deep surgical excision serves as first-line therapy for cSCCs. When performed in earlier stages of disease, this intervention may be successful. However, as standard first-line therapy, surgical outcomes are infrequently published, in contrast to complicated, advanced stage cSCCs that are refractory to surgical intervention and require systemic therapies. Therefore, the true benefit of surgical intervention may be greater than reported in our analysis.

Poor outcomes were reported in the few patients treated with conventional chemotherapy, supporting current recommendations that the risks may outweigh potential benefits [7]. In many cases, chemotherapy was poorly tolerated and disease progression often occurred, resulting in patient death. However, when chemotherapeutic agents were delivered to tumor cells after electroporation, complete responses were observed in 6 patients (46.2%) [49, 50]. The dimension of cSCCs that responded completely to electrochemotherapy ranged from 3 to 15 cm. These results further support existing recommendations that electrochemotherapy may be a potential treatment for RDEB-cSCCs and should be further evaluated in larger cohorts [7].

Radiotherapy can be complicated by delayed wound healing and skin ulcerations due to exposure to ionizing radiation. However, by delivering radiotherapy in small fractions, patients may better tolerate this therapy. In our systematic review examining 8 patients treated with radiotherapy and known clinical outcomes, only one patient achieved a clinical response. As such, there is insufficient evidence to support radiotherapy as a definitive treatment for RDEB-cSCCs [7].

The limited reports of immunotherapies and anti-EGFR therapy for RDEB SCCs have shown promising results in the treatment of RDEB SCCs. Cemiplimab, a PD-1 inhibitor, approved by the FDA in September 2018 for the treatment of metastatic or locally advanced cSCC for which no curative local treatment options are available, was used in 5 patients. Other PD-1 inhibitors such as nivolumab and pembrolizumab, approved for the management of advanced head and neck SCC in November 2016 and June 2020 respectively, were used in 1 and 2 patients, respectively. Anti-EGFR therapy with cetuximab, approved for late-stage head and neck SCC in November 2011, has also been reported in 12 cases. Rigosertib, a polo-like kinase 1 inhibitor, is currently being studied for RDEB-cSCCs with promising outcomes [58–60].

As shown in Fig. 2, our analysis suggests that the addition of immunotherapy and/or anti-EGFR therapies may extend patient survival when first-line surgical management options have been exhausted. It is difficult to determine the added benefit in survival time with immunotherapy and anti-EGFR therapies, as our estimated survival time of 2 years with only surgical management is grossly underestimated due to publication bias favoring more complex cases. Furthermore, these results are limited by the small sample size. Despite the rarity of RDEB, larger cohort studies will be needed to confirm our conclusions and address existing knowledge gaps, including determining the prevalence of PD-1 and EGFR tumor expression in RDEB-cSCCs and correlating increased expression with response to immunotherapy or anti-EGFR therapy, respectively. If such molecular markers are discovered to be common, clinical practice may be modified to include PD-L1 and EGFR staining, alongside routine histologic assessment. In the phase 2 study Keynote-055, in which patients with head and neck cSCC refractory to platinum and cetuximab were treated with pembrolizumab, no significant differences in response rates were observed between those with ≥ 50% PD-1 expression (i.e. percentage of tumor and mononuclear inflammatory cells within tumor nests and adjacent supporting stroma expressing PD-L1 at any intensity) and < 50% PD-1 expression (27% [95%CI 15–42] vs. 13% [95%CI 7–20], respectively) [61]. However, with further stratification of PD-1 expression, a positive prognostic relationship may become clear. Similar studies should also be conducted with anti-EGFR therapy to determine a threshold for EGFR expression that predicts improved response rates and clinical benefit. While these therapies can potentially extend patient survival, their low availability, cost, and poor tolerability, including frequent immune-mediated side effects, are challenges that contribute to their use as a last resort for late-stage cSCCs.

Several limitations of our analysis must be acknowledged. Firstly, existing literature on RDEB-cSCCs primarily consists of case reports and series, which are biased towards novel or favorable outcomes. Furthermore, these literary sources often did not report the time or age of a patient’s cSCC diagnosis or time or age of death, since written while patients are still alive, limiting the availability of patient survival data based on treatment. As such, long-term responses are unclear, including related to the use of more recently introduced EGFR and PD-1 inhibitors with their early good outcomes, limiting generalizable conclusions. Secondly, multiple therapeutic modalities are often used to treat cSCCs. Therefore, we could not determine benefits as such extended survival to a single therapy. Thirdly, literary sources included in our systematic review varied in time of publication (from 1969 to 2022). Consequently, fewer years of patient survival were reported in older literature when modern therapeutic modalities were unavailable.

Even with evidence for efficacy of immunotherapies and anti-EGFR therapies, these drugs are not readily available for RDEB patients due to administrative hurdles. In many cases, their use will remain off-label and health insurances may be hesitant to cover their significant costs, given the life-threatening course of RDEB and high risk of additional aggressive cSCCs. Results from this analysis provide evidence for improving individual treatment decisions in late-stage RDEB-cSCC. At the same time, all possible efforts should be made to improve early detection of RDEB-cSCC, reducing the need for advanced therapies.

## Conclusions

In summary, the majority of RDEB-cSCCs are well-differentiated, ulcerated, and at least 2 cm in size with the most frequent localization to the distal upper and lower extremities. Our analysis of treatment regimens for RDEB-cSCCs suggests that when surgical treatment options have been exhausted, the addition of immunotherapy and/or anti-EGFR therapies may extend patient survival. Use of immunotherapies and anti-EGFR therapies as neoadjuvant therapies should also be explored. A recent phase II study in non-EB patients with resectable stage II to IV (M0) cSCC treated with cemiplimab as neoadjuvant therapy prior to surgery demonstrated an objective response on imaging in 54 patients (68%; 95% CI, 57 to 78) [62]. In total, five achieved a complete response and 49 a partial response [62].

## Methods

### Eligibility criteria

A systematic literature review of PubMed (NLM/NIH), Embase (Elsevier), ClinicalTrials.gov, European Union Clinical Trials Registry, and Cochrane Central Register of Controlled Trials (Wiley) databases was conducted from inception to February 15, 2024 and followed PRISMA guidelines. Our original protocol was published on PROSPERO (CRD42022309377).

A search strategy was defined (see Supplementary Table 2, Additional file 1). Database records were collated and de-duplicated in EndNote and uploaded to Rayyan [63] for screening. Articles included pathophysiology and/or therapies for RDEB and cSCC without restrictions on publication year. Review articles, books, editorials, and non-English text manuscripts were excluded.

#### Screening

All articles were independently screened by at least two authors (AH, ART, AK). Screening was conducted in two rounds: first by title and abstract, then by full text. If a consensus was not reached, a fourth author (AP) was included to make a final determination.

#### Data extraction

Data extraction was performed independently by two authors (AH, AK). Information related to patient demographics and clinical characteristics of RDEB and cSCCs were extracted. Cases that were suspected as duplicates (ie, identical age, treatment regimen, location of cSCCs) were removed. Cases with systemic therapies and unpublished outcomes regarding patient vital status were tracked with corresponding authors via e-mail; of which, 5 of 9 authors responded with patient survival data. Data is available through request to the investigators.

#### Risk of bias assessment

The Joanna Briggs Institute tool [64], Cochrane’s Risk of Bias 2 tool [65], and U.S. National Toxicology Program’s Office of Health Assessment tool [66] were used to identify possible risk of bias in case reports/series and cohort studies, clinical trials, and all other studies, respectively. Overall, the risk of bias was low (see Supplementary Table 3–5, Additional file 1).

#### Statistical analysis

The impact of therapies on patient survival time, defined here as the timeframe between first cSCC diagnosis to death, was determined by categorizing each patient by their treatment regimen. Kaplan–Meier-diagrams were compiled using GraphPad Prism (version 9.4.1, GraphPad Software, San Diego, California USA, www.graphpad.com) to depict survival times with different therapies. Linear regression analysis was performed using Excel (version 2304, Microsoft Software, Redmond, Washington USA, www.microsoft.com) to determine the relationship between age at diagnosis and patient survival. No further statistical analysis could be done due to the heterogeneity of the studies.

### Supplementary Information

**Additional file 1: Supplemental Table 1.** Patients in Survival Curve. **Supplemental Table 2.** Database Search Strategy. **Supplemental Table 3.** Risk of Bias Assessments for Case Reports included in Analysis. **Supplemental Table 4.** Risk of Bias Assessments for Case Series included in Analysis. **Supplemental Table 5.** Risk of Bias Assessments for Cohort Studies included in Analysis.

## Data Availability

The datasets supporting the conclusions of this article are available in the PubMed, Embase, Cochrane, ClinicalTrials.gov, and EudraCT databases: https://pubmed.ncbi.nlm.nih.gov/, https://www.embase.com/, https://clinicaltrials.gov/, https://www.clinicaltrialsregister.eu/ctr-search/search, and https://www.cochranelibrary.com/central.

## Abbreviations

Amp: Amputation
Cem: Cemiplimab
Cet: Cetuximab
CHT: Unspecified chemotherapy
cSCC: Cutaneous squamous cell carcinoma
ECT: Electrochemotherapy
EGFR: Epidermal growth factor
Erl: Erlotinib
Exc: Excision
5-FU: 5-Fluorouracil
Gem: Gemcitabine
Int: Intermediate RDEB
Meta: Metastases
MTX: Methotrexate
Niv: Nivolumab
NR: No response
Pan: Panitumumab
PD: Progressive disease
Pem: Pembrolizumab
PR: Partial response
RC/RL: Local recurrence or relapse
RDEB: Recessive dystrophic epidermolysis buillosa
RDEB-Int/I: Intermediate RDEB
RDEB-Sev/S: Severe RDEB
RT: Radiotherapy
SCC: Squamous cell carcinoma
SD: Stable disease
Sev: Severe RDEB
SX: Surgery
T-VEC: Talimogene laherparepvec
-: Not applicable as patients only underwent surgery for their treatment
