# Supplementary material for: Therapies for cutaneous squamous cell carcinoma in recessive dystrophic epidermolysis bullosa: a systematic review of 157 cases
Source: Orphanet J Rare Dis. 2024 May 21;19:206. doi: 10.1186/s13023-024-03190-1 (PMC11106883; doi:10.1186/s13023-024-03190-1)
Supplement: Supplementary file 1 — Additional file 1: Supplemental Table 1. Patients in Survival Curve. Supplemental Table 2. Database Search Strategy. Supplemental Table 3. Risk of Bias Assessments for Case Reports included in Analysis. Supplemental Table 4. Risk of Bias Assessments for Case Series included in Analysis. Supplemental Table 5. Risk of Bias Assessments for Cohort Studies included in Analysis. [file 13023_2024_3190_MOESM1_ESM.docx]

**Supplemental Table 1: Patients in Survival Curve**

| Reference | Age at 1^st^ SCC Diagnosis (yr)/Sex | RDEB Subtype | Total SCCs during patient lifetime | Site(s) of SCC under Treatment | Histological Differentiation | Site(s) of Metastases | Treatment | Outcomes (if multiple treatments) | Survival time (years), from first SCC diagnosis |
| --- | --- | --- | --- | --- | --- | --- | --- | --- | --- |
| **Surgery only** |  |  |  |  |  |  |  |  |  |
| Robertson 2021 (28) | 27 M | Sev | 2 | Unknown | Unknown | None | Exc | - | 0.5 |
| Rengen 1996 (64) | 46 F | Sev | 4 | Hand | Well | None | Exc+Amp | - | 0.63 |
| Miura 2011 (36) | 20 M | N/A | 1 | Foot | Well | Lungs | Amp | - | 0.8 |
| Robertson 2021 (28) | 26 M | Sev | 1 | Unknown | Unknown | Loco-regional metastasis | Exc | - | 1 |
| Suss 2007 (32) | 27 F | Sev | 2 | Upper arm | Moderate | Lymph nodes, Lungs | Amp | - | 1.08 |
| Robertson 2021 (28) | 23 M | Sev | 1 | Unknown | Unknown | None | Exc | - | 1.2 |
| Robertson 2021 (28) | 51 F | Sev | 4 | Unknown | Unknown | Loco-regional metastasis | Exc | - | 1.2 |
| Robertson 2021 (28) | 40 M | Sev | 1 | Unknown | Unknown | Loco-regional metastasis | Exc | - | 1.8 |
| Kim 2018 (6) | 17 M | Sev | 2 | Unknown | Unknown | Metastasis, unspecified | Amp | - | 2 |
| Wechsler 1970 (44) | 22 F | Sev | NR | Hand | Unknown | Axillary lymph nodes | Exc+Amp | - | 2 |
| Bosch 1999 (65) | 30 M | N/A | 3 | Hand, Lower leg | Well | Inguinal lymph nodes | Amp | - | 2.3 |
| Robertson 2021 (28) | 50 F | Sev | 2 | Unknown | Unknown | Metastasis, unspecified | Exc | - | 2.4 |
| Robertson 2021 (28) | 29 F | Sev | 3 | Unknown | Unknown | None | Exc | - | 3.3 |
| Kim 2018 (6) | 19 F | Sev | 8 | Unknown | Unknown | Metastasis, unspecified | Amp | - | 4 |
| Wechsler 1970 (44) | 56 M | Sev | 1 | Hand | Unknown | None | Exc | - | 4 |
| Robertson 2021 (28) | 35 F | Sev | 8 | Unknown | Unknown | Distant metastases | Amp | - | 4.8 |
| Kim 2018 (6) | 20 F | Sev | 1 | Unknown | Unknown | Metastasis, unspecified | Amp | - | 5 |
| Georgeu 2002 (26) | 31 M | N/A | 2 | Upper leg, Lower leg | Unknown | Inguinal lymph nodes, Lungs | Exc | - | 5 |
| Robertson 2021 (28) | 22 F | Sev | 5 | Unknown | Unknown | Loco-regional metastasis | Exc | - | 6 |
| **Surgery +Chemotherapy** |  |  |  |  |  |  |  |  |  |
| Lentz 1990 (37) | 22 F | N/A | 3 | Hand, Forearm | Well | Axillary lymph nodes, Lungs | 1. Exc+Amp  2. Cisplatin | 1. RC/RL  2. Death from metastases | 0.92 |
| Lopes 2020 (33) | 25 F | N/A | NR | Hand | Moderate | Axillary lymph nodes | 1. Amp  2. CHT | 1. Meta  2. Death from SCC | 1 |
| Robertson 2021 (28) | 22 F | Sev | 1 | Unknown | Unknown | Loco-regional metastasis | 1. Exc  2. ECT | 1. RC/RL  2. Death from SCC | 1.6 |
| Robertson 2021 (28) | 32 M | Sev | 5 | Unknown | Unknown | Loco-regional metastasis | 1. Exc  2. CHT | 1. RC/RL  2. Death from SCC | 2.1 |
| Robertson 2021 (28) | 22 M | Sev | 2 | Unknown | Unknown | Loco-regional metastasis | 1. Exc  2. Imiquimod  3. Systemic Retinoid  4. ECT | 1. RC/RL  2. Unknown  3. Unknown  4. Death from SCC | 2.4 |
| Robertson 2021 (28) | 42 M | Sev | 17 | Unknown | Unknown | Loco-regional metastasis | 1. Exc  2. CHT | 1. RC/RL  2. Death from SCC | 12.6 |
| **Surgery**  **+Radiotherapy** |  |  |  |  |  |  |  |  |  |
| McGrath 1991 (49) | 48 F | Sev | 3 | Forearm, Hand | Unknown | Axillary lymph nodes | 1. Exc  2. RT | 1. Meta  2. Death from metastases | 0.5 |
| Dammak 2009 (66) | 18 F | Sev | 1 | Upper leg | Well | Inguinal lymph nodes | 1. Exc  2. RT | 1. RC/RL  2. Death from infection | 1.25 |
| Robertson 2021 (28) | 23.1 F | Sev | 2 | Unknown | Unknown | Loco-regional metastasis | 1. Exc  2. RT | 1. RC/RL  2. Death from SCC | 1.3 |
| Keefe 1988 (48) | 35 F | NR | 2 | Hand | Well | Axillary and cervical lymph nodes, Lungs, Adrenal glands, Thoracic vertebrae | 1. RT  2. Amp | 1. PR  2. Death from SCC | 1.33 |
| Kim 2018 (6) | 36 M | Int | 9 | Unknown | Unknown | Metastasis, unspecified | 1. Exc  2. RT | 1. RC/RL  2. Death | 4 |
| Robertson 2021 (28) | 23.6 M | Sev | 9 | Unknown | Unknown | Loco-regional metastasis | 1. Exc  2. RT | 1. RC/RL  2. Death from SCC | 5 |
| Robertson 2021 (28) | 28.5 F | Sev | 6 | Unknown | Unknown | Loco-regional metastasis | 1. Exc  2. RT | 1. RC/RL  2. Death from SCC | 8.5 |
| Robertson 2021 (28) | 36.2 F | Sev | 22 | Unknown | Unknown | Loco-regional metastasis | 1. Exc  2. RT | 1. RC/RL  2. Death from SCC | 10 |
| Kim 2018 (6) | 39 M | Int | 8 | Unknown | Unknown | Metastasis, unspecified | 1. Exc  2. RT | 1. RC/RL  2. Death | 12 |
| **Surgery +Chemotherapy +Radiotherapy** |  |  |  |  |  |  |  |  |  |
|  |  |  |  |  |  |  |  |  |  |
| Wechsler 1970 (44) | 41 M | Sev | 1 | Foot | Unknown | Lungs | 1. Exc  2. RT  3. MTX | 1. RC/RL  2. Unknown  3. Death from metastases | 6 |
| Wechsler 1970 (44) | 33 M | Sev | 2 | Forearm | Well | Axillary lymph nodes | 1. RT  2. Exc+Amp  3. 5-FU | 1. PR  2. RC/RL  3. Death from renal failure | 13 |
| **+Immunotherapy** |  |  |  |  |  |  |  |  |  |
| Duong 2021 (22) | 27 F | NR | 5 | Back | Well | None | 1. Exc  2. MTX  3. Cem | 1. PD  2. PR  3. SD | 4 |
| Medek 2019 (19) | 29 F | NR | 1 | Forearm | Unknown | Axillary and infra-clavicular lymph nodes | 1. Exc  2. Cet  3. ECT + MTX  4. Pem + T-VEC + Pan | 1. Meta  2. PR  3. PR  4. Death from SCC | 4.1 |
| O’Sullivan 2020 (27) | 28 F | NR | 2 | Chest | Unknown | Unknown | 1. Debulking surgery  2. ECT  3. Cem | 1. PR  2. PR  3. SD | 5 |
| Trefzer 2023 (52) | 36 M | N/A | 5 | Head, Hand | Well | None | 1. Exc  2. Cem+RT | 1. PD  2. SD | 6 |
| **+Anti-EGFR Therapy** | |  |  |  |  |  |  |  |  |
| Diociaiuti 2019 (18) | 15 F | Sev | 1 | Upper arm | Well | Axillary and clavicular lymph nodes | 1. ECT  2. Cet | 1. PD  2. Death from SCC | 0.375 |
| Robertson 2021 (28) | 18 F | Sev | 5 | Unknown | Unknown | Loco-regional metastasis, unspecified | 1. Exc  2. RT  3. Systemic Retinoid  4. Erl | Unknown | 1.4 |
| Arnold 2009 (15) | 23 F | Sev | 3 | Elbow, Feet | Well | Axillary lymph nodes | 1. Exc  2. RT  3. CHT  4. Cet | 1. RC/RL and Meta  2. PD  3. PR  4. SD | 2 |
| Robertson 2021 (28) | 13 M | Sev | 2 | Unknown | Unknown | Loco-regional metastasis, unspecified | 1. Exc  2. Cet | 1. RC/RL  2. Death from SCC | 3.8 |
| Diociaiuti 2019 (18) | (?) F | Sev | NR | Lower leg | Poor | Axillary and para-iliac lymph nodes | 1. Exc  2. Cet  3. Amp  4. RT | 1.RC/RL and Meta  2. PR  3. RX/RL  4. Death from SCC | 3.9 |
| Kim 2018 (6) | 30 M | Sev | 7 | Unknown | Unknown | Metastasis, unspecified | 1. Exc  2. Acitretin  3. CHT  4. Cet | Unknown | 4 |
| Trefzer 2023 (52) | 16 M | N/A | 3 | Forearm, Foot | Well | Inguinal lymph nodes | 1. Exc  2. Cem  3. Amp  4. Cet | 1. PD  2. PD  3. SD  4. PD | 12 |
| Kim 2018 (6) | 39 M | Int | 8 | Unknown | Unknown | Metastasis, unspecified | 1. Amp  2. RT  3. Acitretin  4. CHT  5. Cet | Unknown | 13 |
| Kim 2018 (6) | 16 F | Sev | 10 | Unknown | Unknown | Metastasis, unspecified | 1. Amp  2. RT  3. Acitretin  4. Cet | Unknown | 13 |
| Reimer 2020 (21) | 25 F | Sev | >80 | Forearm, Hand, Knee | Unknown | Inguinal lymph nodes | 1. Amp  2. Pem  3. Cet | 1. Meta  2. PD  3. Death | 26.75 |

Amp, amputation; Cem, Cemiplimab; Cet, Cetuximab; CHT, unspecified chemotherapy; ECT, electrochemotherapy; Erl, Erlotinib; Exc, excision; 5-FU, 5-fluorouracil; Gem, Gemcitabine; Int, intermediate RDEB; Meta, metastases; MTX, methotrexate; Niv, Nivolumab; NR, no response; Pan, panitumumab; PD, progressive disease; Pem, Pembrolizumab; PR, partial response; RC/RL, local recurrence or relapse; RT, radiotherapy; SD, stable disease; Sev, severe RDEB; T-VEC, talimogene laherparepvec; -, not applicable as patients only underwent surgery for their treatment

Supplemental Table 2: Database Search Strategy

| **PubMed (NLM/NIH):**  ("Epidermolysis Bullosa"[Mesh] OR "Epidermolysis Bullosa") AND ("Carcinoma, Squamous Cell"[Mesh] OR "Carcinoma") |
| --- |
| **Embase (Elsevier):**  (epidermolysis bullosa'/exp OR 'epidermolysis bullosa' OR 'epidermolysis bullosa dystrophica'/exp OR 'dystrophic epidermolysis bullosa' OR 'epidermolysis bullosa dystrophica') AND (‘squamous cell carcinoma'/exp OR 'cancer, squamous cell' OR 'carcinoma, squamous cell' OR 'epidermoid cancer' OR 'epidermoid carcinoma' OR 'epidermoid cell carcinoma' OR 'intra epithelial epidermoid carcinoma' OR 'neoplasms, squamous cell' OR 'planocellular carcinoma' OR 'prickle cell cancer' OR 'prickle cell carcinoma' OR 'squamous carcinoma' OR 'squamous cell cancer' OR 'squamous cell carcinoma' OR 'squamous cell epithelioma' OR 'squamous cell neoplasms' OR 'squamous epithelioma' OR 'squamous epithelium carcinoma' OR ‘skin carcinoma'/exp OR 'skin carcinoma') |
| Clinicaltrials.gov  “Epidermolysis Bullosa” AND “Squamous Cell Carcinoma” |
| EudraCT  “Epidermolysis Bullosa” AND “Squamous Cell Carcinoma” |
| Cochrane Central Register of Controlled Trials (Wiley)  (“Epidermolysis Bullosa” OR MeSH descriptor: [Epidermolysis Bullosa] explode all trees) AND (“Squamous Cell Carcinoma” OR MeSH descriptor: [Carcinoma, Squamous Cell] explode all trees) |
| No time constraints or filters were applied |

**Supplemental Table 3: Risk of Bias Assessments for Case Reports included in Analysis**

| **Study** | **Q1** | **Q2** | **Q3** | **Q4** | **Q5** | **Q6** | **Q7** | **Q8** | **Overall Risk of Bias** |
| --- | --- | --- | --- | --- | --- | --- | --- | --- | --- |
| Trefzer 2023 | Y | Y | Y | Y | Y | Y | Y | Y | Low |
| Vasilev 2022 | Y | Y | Y | Y | Y | Y | Y | Y | Low |
| Duong 2021 | Y | Y | Y | Y | Y | Y | Y | Y | Low |
| Lopes 2020 | Y | Y | Y | Y | Y | Y | Y | Y | Low |
| Reimer 2020 | Y | Y | Y | Y | Y | Y | Y | Y | Low |
| Piccerillo 2020 | Y | Y | Y | Y | Y | Y | Y | Y | Low |
| Khaddour 2020 | Y | Y | Y | Y | Y | Y | Y | Y | Low |
| Saito 2019 | Y | Y | Y | Y | Y | Y | Y | Y | Low |
| Medek 2019 | Y | Y | Y | Y | Y | Y | Y | Y | Low |
| Strupp 2017 | Y | N | Y | N | Y | N | N | Y | Unclear |
| Hata 2015 | Y | Y | Y | Y | Y | Y | N | Y | Low |
| Chaptini 2015 | Y | Y | Y | Y | Y | Y | N | Y | Low |
| Ng 2014 | Y | N | Y | N | Y | Y | N | Y | Unclear |
| Sakan 2013 | Y | Y | Y | Y | Y | Y | Y | Y | Low |
| Tsukada 2012 | Y | Y | Y | Y | Y | Y | N | Y | Low |
| Larocca 2012 | Y | Y | Y | Y | Y | N | Y | Y | Low |
| Huang 2011 | Y | Y | Y | N | Y | N | N | Y | Unclear |
| Miura 2011 | Y | Y | Y | Y | Y | Y | Y | Y | Low |
| Inverardi 2011 | Y | Y | Y | N | Y | N | N | Y | Unclear |
| Cuadrado-Corrales 2011 | Y | Y | Y | Y | Y | Y | N | Y | Low |
| Rodriguez-Lojo 2011 | Y | Y | Y | Y | Y | Y | Y | Y | Low |
| Shivaswamy 2009 | Y | Y | Y | Y | Y | Y | Y | Y | Low |
| Buonocore 2009 | N | N | N | Y | Y | Y | Y | Y | Unclear |
| Arnold 2009 | Y | Y | Y | Y | Y | Y | Y | Y | Low |
| Rokunohe 2008 | Y | Y | Y | Y | Y | Y | Y | Y | Low |
| Suss 2007 | Y | Y | Y | Y | Y | Y | Y | Y | Low |
| Saxena 2006 | Y | Y | Y | Y | Y | Y | Y | Y | Low |
| Perez-Naranjo 2005 | Y | Y | Y | Y | Y | Y | Y | Y | Low |
| Souza 2005 | Y | Y | Y | Y | Y | Y | Y | Y | Low |
| Yamada 2004 | Y | Y | Y | Y | Y | Y | Y | Y | Low |
| Tomita 2003 | Y | Y | Y | Y | Y | Y | Y | Y | Low |
| Kawasaki 2003 | Y | Y | Y | Y | Y | Y | Y | Y | Low |
| Kalisiak 2003 | Y | Y | Y | Y | Y | Y | Y | Y | Low |
| Ayman 2002 | Y | Y | Y | Y | Y | Y | Y | Y | Low |
| Hosokawa 1998 | Y | Y | Y | Y | Y | N | N | Y | Low |
| Rengen 1996 | Y | Y | Y | Y | Y | Y | N | Y | Low |
| Chorny 1993 | Y | N | Y | Y | Y | N | N | Y | Unclear |
| Whitney 1993 | Y | Y | Y | Y | Y | Y | Y | Y | Low |
| Schreiber 1993 | Y | Y | Y | N | Y | N | Y | Y | Low |
| McGrath 1991 | Y | Y | Y | Y | Y | N | N | Y | Low |
| McGrath 1991 | Y | Y | Y | Y | Y | Y | Y | Y | Low |
| Keefe 1988 | Y | Y | Y | Y | Y | Y | Y | Y | Low |
| Callen 1987 | Y | Y | Y | Y | Y | Y | Y | Y | Low |
| Cardoso 1986 | Y | Y | Y | Y | Y | Y | Y | Y | Low |
| Tidman 1984 | Y | Y | Y | Y | Y | Y | Y | Y | Low |
| Carapeto 1982 | Y | Y | Y | Y | Y | Y | Y | Y | Low |
| Edland 1969 | Y | Y | Y | Y | Y | Y | Y | Y | Low |

Key: Y, yes; N, no

Q1 Were patient’s demographic characteristics clearly described?

Q2 Was the patient’s history clearly described and presented as a timeline?

Q3 Was the current clinical condition of the patient on presentation clearly described?

Q4 Were diagnostic tests or assessment methods and the results clearly described?

Q5 Was the intervention(s) or treatment procedure(s) clearly described?

Q6 Was the post-intervention clinical condition clearly described?

Q7 Were adverse events (harms) or unanticipated events identified and described?

Q8 Does the case report provide takeaway lessons?

**Supplemental Table 4: Risk of Bias Assessments for Case Series included in Analysis**

| **Study** | **Q1** | **Q2** | **Q3** | **Q4** | **Q5** | **Q6** | **Q7** | **Q8** | **Q9** | **Q10** | **Overall Risk of Bias** |
| --- | --- | --- | --- | --- | --- | --- | --- | --- | --- | --- | --- |
| Diociaiuti 2019 | Y | Y | Y | - | - | Y | Y | Y | - | Y | Low |
| Diociaiuti 2016 | Y | Y | Y | - | - | Y | Y | Y | - | Y | Low |
| Dammak 2009 | Y | Y | Y | - | - | Y | Y | Y | - | Y | Low |
| Georgeu 2002 | Y | Y | Y | - | - | Y | Y | Y | - | Y | Low |
| Weber 2001 | Y | Y | Y | - | - | Y | Y | Y | - | Y | Low |
| Bosch 1999 | Y | Y | Y | - | - | Y | Y | Y | - | Y | Low |
| Newman 1992 | Y | Y | Y | - | - | Y | Y | Y | - | Y | Low |
| McGrath 1992 | Y | Y | Y | - | - | Y | Y | Y | Y | Y | Low |
| Yoshioka 1991 | Y | Y | Y | - | - | Y | Y | Y | - | Y | Low |
| Lentz 1990 | Y | Y | Y | - | - | Y | Y | Y | - | Y | Low |
| Reed 1975 | Y | - | - | - | - | Y | Y | N | - | Y | High |
| Wechsler 1970 | Y | - | - | - | - | Y | Y | - | - | Y | High |

Y, yes; N, no; -, unclear

Q1 Were there clear criteria for inclusion in the case series?

Q2 Was the condition measured in a standard reliable way for all participants included in the case series?

Q3 Were valid methods used for identification of the condition for all participants included in the case series?

Q4 Did the case series have consecutive inclusion of participants?

Q5 Did the case series have complete inclusion of participants?

Q6 Was there clear reporting of the demographics of the participants in the study?

Q7 Was there clear reporting of clinical information of the participants?

Q8 Were the outcomes or follow up results of cases clearly reported?

Q9 Was there clear reporting of the presenting site(s)/clinic(s) demographic information?

Q10 Was statistical analysis appropriate?

**Supplemental Table 5: Risk of Bias Assessments for Cohort Studies included in Analysis**

| **Study** | **Q1** | **Q2** | **Q3** | **Q4** | **Q5** | **Q6** | **Q7** | **Q8** | **Q9** | **Q10** | **Q11** | **Overall Risk of Bias** |
| --- | --- | --- | --- | --- | --- | --- | --- | --- | --- | --- | --- | --- |
| Paganelli 2022 | Y | Y | Y | - | - | Y | Y | - | - | - | Y | Unclear |
| Filoni 2020 | Y | Y | Y | - | - | - | Y | - | - | - | Y | Unclear |
| Bartola 2020 | Y | Y | Y | - | - | Y | Y | Y | Y | - | Y | Low |
| Robertson 2020 | Y | Y | Y | - | - | Y | Y | Y | Y | Y | Y | Low |
| Castelo 2019 | Y | Y | Y | - | - | Y | Y | Y | Y | - | Y | Low |
| Kim 2018 | Y | Y | Y | - | - | Y | Y | Y | Y | Y | Y | Low |
| Fine 2009 | Y | Y | Y | Y | - | Y | Y | Y | Y | Y | Y | Low |

Y, yes; N, no; -, unclear

Q1 Were the two groups similar and recruited from the same population?

Q2 Were the exposures measured similarly to assign people to both exposed and unexposed groups?

Q3 Was the exposure measured in a valid and reliable way?

Q4 Were confounding factors identified?

Q5 Were strategies to deal with confounding factors stated?

Q6 Were the groups/participants free of the outcome at the start of the study (or at the moment of exposure)?

Q7 Were the outcomes measured in a valid and reliable way?

Q8 Was the follow up time reported and sufficient to be long enough for outcomes to occur?

Q9 Was follow up complete, and if not, were the reasons to loss to follow up described and explored?

Q10 Were strategies to address incomplete follow up utilized?

Q11 Was appropriate statistical analysis used?
